# Primary Ovarian Choriocarcinoma: Rare Entity

**DOI:** 10.1155/2021/4545375

**Published:** 2021-09-24

**Authors:** Mequanent Tariku Adow, Shimelis Fantu Gebresilasie, Natnael Alemayehu Abebe

**Affiliations:** ^1^Debre Tabor University, College of Health Science, Debre Tabor, Ethiopia; ^2^Hawassa University, College of Medicine and Health Science, Hawassa, Ethiopia

## Abstract

**Background:**

Primary pure ovarian choriocarcinoma is a rare aggressive tumor which can be nongestational arising from germ cells or gestational origin. Preoperative diagnosis of extrauterine choriocarcinoma is challenging due to nonspecific clinical presentation. *Case Presentation*. This article reports primary ovarian choriocarcinoma, likely gestational in a 25-year-old para 2 woman presenting with lower abdominal pain and swelling of two-week duration. Diagnosis was suspected by serum beta-human chorionic gonadotropin and confirmed histologically after surgery. Postoperatively, she was managed with multiple courses of chemotherapy using a bleomycin, etoposide, and cisplatin regimen, and the treatment was effective.

**Conclusion:**

In patients with adnexal mass presenting with nonspecific symptoms especially with high Doppler blood flow of the mass on ultrasound evaluation, serum beta-human chorionic gonadotropin determination is recommended before laparotomy. In setups where the genomic test is not available, histological and clinical effort to differentiate gestational versus nongestational choriocarcinoma is useful for specific management decision.

## 1. Background

Primary pure ovarian choriocarcinoma is a highly aggressive tumor which arises from ovarian ectopic pregnancy as gestational choriocarcinoma or as nongestational choriocarcinoma from germ cells [1]. The estimated incidence of pure nongestational ovarian carcinoma is 1 : 369 million. Primary extrauterine gestational choriocarcinoma has prevalence of 1 : 5,335 ovarian pregnancies and 1 : 2.2 million normal intrauterine pregnancies which makes primary gestational ovarian origin even rarer [2]. Due to rarity of primary ovarian choriocarcinoma and nonspecific clinical presentation, reaching diagnosis of gestational versus nongestational origin and treatment selection is challenging.

## 2. Case Presentation

A 25-year-old para 2 woman with last normal menstrual period three weeks back presented with lower abdominal swelling and pain of two-week duration. She has associated loss of appetite but no cough, shortness of breath, or vaginal bleeding. Her last delivery was 2 years back. She was not taking any form of contraception.

On physical examination, she was acutely sick looking in pain with blood pressure of 130/80 mmHg, pulse rate of 108 beats per minute, respiratory rate of 22 breaths per minute, and temperature of 37.1 degree centigrade. Further examination revealed fourteen-week-sized mildly tender, firm, mobile abdominopelvic mass, bulged posterior vaginal fornix, and normal cervix.

Ultrasound showed huge multiseptated adnexal mass with internal solid component, likely originating from the left ovary. The solid component and septum had blood flow on the Doppler study. There was also free peritoneal fluid. On chest the X-ray film, the costophrenic angle was blunted with pleural effusion.

Her blood group was B-ve and had hematocrit of 29%, platelet of 768,000, negative urine human chorionic gonadotropin (hCG), and normal liver and renal function tests. Some available tumor markers were also determined: CA-125 (189.6 U/mL) and LDH (1044 U/mL).

**Figure 1:**
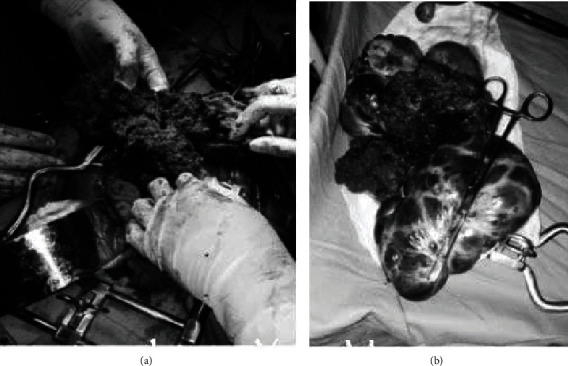
Gross appearance of choriocarcinoma intraoperatively (a) and with cyst after excision (b).

Exploratory laparotomy was decided with impression of the malignant ovarian tumor. Intraoperatively, about 200 mL bloody ascetic fluid was found in the peritoneal cavity together with 12 × 14 cm fragile, dark left ovarian mass attached to the uterine serosa and dome of the urinary bladder. There was also 10 × 6 cm cystic ipsilateral ovarian mass adjacent to the fragile tumor (Figure 1).

Hemorrhagic fluid was sucked out, and the mass was excised with part of the dome of the bladder. Total abdominal hysterectomy and bilateral salpingo-oophorectomy were performed, and the bladder was repaired.

**Figure 2:**
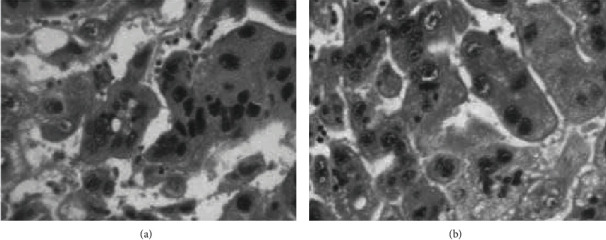
Microscopic section showing highly pleomorphic trophoblast cells with irregular, hyperchromatic prominent nuclei and mitotic activity with areas of extensive hemorrhage (a and b).

All the tissues were submitted for histopathology and revealed solid proliferation of round to oval pleomorphic cells exhibiting an indistinct eosinophilic cytoplasm, coarse nuclear chromatin, prominent nucleoli, abundant multinucleate giant cells, extensive necrosis, and hemorrhage (Figure 2).

A section from the cyst structure showed a fibrous convoluted cyst wall, cavity with calcification, and luteinized stroma. Sections from the uterus, cervix, and tubes showed normal appearance. No evidence of other germ cell element was noticed. The patient was stable postoperatively, and serum B-hCG was determined to be 1,000,000 mIU/mL.

After three weeks of surgery, the patient received multiple courses of the bleomycin, etoposide, and cisplatin (BEP) regimen. The posttherapeutic period was smooth, and serum B-hCG becomes normal. Currently, the patient is at the 12^th^ month of follow-up being disease free and having no evidence of recurrence or metastasis.

## 3. Discussion and Conclusions

Ovarian choriocarcinoma is a rare but highly aggressive tumor. This may arise from metastatic gestational choriocarcinoma from the uterus or tubes or as primary ovarian origin [3]. Pure choriocarcinoma has been defined as a tumor that does not include other germ cell tumor elements [4]. Primary pure ovarian choriocarcinoma is a rare neoplasm which could be of nongestational or gestational origin. Pure nongestational choriocarcinoma is of germ cell origin with estimated incidence of 1 : 369,000,000. Primary extrauterine gestational choriocarcinoma is rare and has prevalence of 1 : 5,335 ovarian pregnancies and 1 : 2.2 million normal intrauterine pregnancies [2]. As to the reading of the researchers, there is no estimated prevalence of choriocarcinoma primarily originating from ovarian gestation.

Abdominal pain, detection of abdominopelvic mass, abnormal vaginal bleeding, or metastatic manifestations to the lung and brain could be the nonspecific presentation. Lesions which appear before menarche manifest with isosexual precocious puberty in about 50% of patients [5]. These nongestational and gestational choriocarcinomas are difficult to differentiate based on history, clinical presentation, or histology. Historically, nongestational choriocarcinoma may be suspected if it occurs before menarche or if one never had sexual intercourse. Gestational choriocarcinoma is more sensitive than nongestational choriocarcinoma for chemotherapy, and the treatment regimen is different. Appropriate differentiation between the two forms by using DNA genomic testing to see the presence of a paternal gene is preferred for management [6, 7]. This technique is expensive and not available in our setups. In the absence of DNA analysis, the morphological presence of a well-developed corpus luteum of pregnancy adjacent to the tumor may be indicative of a gestational origin [8]. Due to this finding and the fact that she is in a reproductive age with no any form of contraception and history of delivery two years back added with very high level of serum B-hCG, gestational primary ovarian choriocarcinoma is likely.

Confirmed extrauterine gestational choriocarcinoma can be managed with methotrexate-based chemotherapy reserving excision of the local tumor and postoperative chemotherapy for a bulky or chemoresistant tumor. Confirmed nongestational choriocarcinoma preoperatively or intraoperatively in young patients, who desire their fertility, could be managed with comprehensive staging fertility preserving surgery. Lymphadenectomy was considered with suspected nodal metastasis [5, 9]. In postmenopausal women and in patients with an advanced-stage disease or with bilateral ovarian involvement, hysterectomy and bilateral salpingo-oophorectomy could be performed with careful surgical staging. Chemotherapy with the BEP regimen becomes the standard management for ovarian germ cell malignancies due to high response rate and fertility preservation with healthy offspring [10]. This regimen is used to treat gestational trophoblastic neoplasm including resistant cases for methotrexate-based management [11, 12]. In this case, optimal debulking surgery was planned for an advanced ovarian tumor and choriocarcinoma was considered from intraoperative gross finding. Preoperatively, urine hCG was negative likely due to a hook effect [13]. Had there been strong suspicion, this effect could be avoided by making multiple dilutions or sending a sample for assays which can detect much degraded molecules [13, 14]. Immediately after the operation, the serum B-hCG was determined to be 1,000,000 mIU/mL. This hormone is important not only for diagnostic purposes but also for checking treatment response during follow-up. Had it been diagnosed preoperatively, at least unnecessary right oophorectomy could have been avoided in this young patient.

Preoperative diagnosis of extrauterine choriocarcinoma is challenging due to nonspecific clinical presentation. In the present case, ovarian choriocarcinoma was diagnosed after surgery with histology and serum B-hCG. Primary ovarian choriocarcinoma is an extremely rare aggressive tumor. In patients with adnexal mass especially high Doppler blood flow, serum B-hCG determination is recommended before laparotomy. In setups where the genomic test is not available, histological and clinical effort to differentiate gestational versus nongestational choriocarcinoma is useful for specific management decision.

## Data Availability

A copy of the case clinical information, informed consent form, and images is available for review by the editor of this journal.
